# Erenumab in the Treatment of Comorbid Trigeminal Neuralgia in Patients With Migraine

**DOI:** 10.7759/cureus.35913

**Published:** 2023-03-08

**Authors:** Masahito Katsuki, Shin Kawamura, Kenta Kashiwagi, Senju Tachikawa, Akihito Koh

**Affiliations:** 1 Department of Neurosurgery, Itoigawa General Hospital, Itoigawa, JPN; 2 Department of Neurology, Itoigawa General Hospital, Itoigawa, JPN

**Keywords:** headache, migraine, alternative medicine, trigeminal neuralgia, erenumab, anti-calcitonin gene-related peptide monoclonal antibodies

## Abstract

Background

Surgical treatment for trigeminal neuralgia (TN) sometimes becomes difficult. Erenumab, an anti-calcitonin gene-related peptide (CGRP)-receptor monoclonal antibody, is used for migraine and potentially has efficacy for TN.

Method

We retrospectively investigated six migraine patients with comorbidity treated with 70 mg of erenumab. Monthly headache days and a numerical rating scale (NRS) of TN were evaluated before, one, and three months after erenumab administration.

Results

Before being treated with 70 mg of erenumab, the six migraine patients with comorbid TN had taken at least one sort of preventative medication, but it had been ineffective. During the three-month erenumab use, previous medications were continued. The median age was 71 years (range 59-87). The six patients (five females and one male) had episodic migraine. Three had TN due to vessels, one had TN due to a tumor, one had TN without neurovascular compression, and one had an undetermined etiology. Five (83%) of the six patients reported improved NRS of TN. The median NRS of TN before, one, and three months after treatment were 8 (7-10), 3.5 (0-10), and 2 (0-5, n=4). Monthly headache days were 4 (4-10), 2.5 (0-4), and 1 (1-2, n=4). There were no side effects of erenumab.

Conclusion

Surgical treatment sometimes cannot be performed for those with TN. Our findings were preliminary and a bigger sample size is required for this study to draw firmer conclusions. However, it is possible, although rare, that there are migraine patients for whom the NRS of comorbid TN improves with the use of erenumab, an anti-CGRP receptor monoclonal antibody.

## Introduction

A clinical condition known as trigeminal neuralgia (TN) is characterized by intense pain paroxysms in the trigeminal nerve's somatosensory distribution that is accompanied by hypersensitivity to non-nociceptive stimuli [1-3]. Even the slightest stimulus, such as speaking, eating, or a small touch on the skin, might cause discomfort to start. Even though it is typically unilateral, the pain can happen at random and commonly returns during the day. TN is an uncommon disease that affects 4-13 persons per 100,000 annually. Prevalence ratios of men to women typically hover around 1:1.6. According to conventional wisdom, the trigeminal nerve's compression and structural alterations are the most typical causes of TN. A weakness exists in the "root entry zone" (REZ). The surgical plan tries to correct the trigeminal nerve's morphological alterations, such as distortion, dislocation, and distension, or transpose the problematic vessels from the REZ to other regions [4]. If there are no apparent offending vessels [5] or tumors, internal neurolysis can be considered [6]. These surgical treatments are performed when internal medical treatments such as anticonvulsants and gabapentinoids fail. However, the time to recurrence is shorter after surgery in patients over 60 years [7], and repeated surgery is sometimes needed [8]. There may be cases in which treatment methods become difficult, and palliative care is required due to old age, a decline in surgical tolerance, and difficulty of reoperation.

Calcitonin gene-related peptide (CGRP) is a crucial peptide in the pathophysiology of orofacial pain. In TN and migraine patients, CGRP is increased [9,10]. It is suggested that CGRP generated from intraganglionic areas may affect pain transmission by releasing different cytokines because CGRP is also high in TN patients. Erenumab, an anti-CGRP-receptor monoclonal antibody, is now widely used as a prophylactic medication for migraine [11,12]. Considering that CGRP has some roles in TN physiology, treating TN with CGRP-related monoclonal antibodies can be suggested. Also, an increased risk of TN in those with migraine was reported by Lin et al. [13]. In their study in Taiwan, the incident rate of newly diagnosed TN among migraine cohorts was 136.39/100,000 person-years. The hazard ratio for TN was 6.72 (95% CI, 5.37-8.41; p < 0.001) in migraine cohorts compared to non-migraine cohorts.

Previously, two reports described the efficacy of erenumab for TN [9,10], but similar articles remain few. Here, we report six cases of comorbid TN in patients with migraine as real-world data and an early experience. Our report is the first in terms of case reports for patients with both migraine and TN and suggests that erenumab may be an alternative medicine for TN with comorbid migraine. 

## Materials and methods

Study population

Six consecutive patients with migraine and comorbid TN at our headache-specialized outpatient center between April 2021 and December 2022 were investigated. Our treatment strategy was based on the Clinical Practice Guideline for Headache Disorders 2021. The patients had been keeping headache diaries and had experienced TN and migraine for at least 90 days prior to the erenumab treatment. They had taken at least one form of preventative medicine (valproic acid or Japanese herbal kampo goreisan [14]) before erenumab use. However, the prophylactic medication failed. Therefore, they were treated with 70 mg erenumab every 28 days, not 140 mg. 

A guideline exists in Japan for the administration of erenumab. All of the patients in this report followed it. The guidelines indicate that patients must: (i) have multiple migraine attacks per month with or without aura, or have been diagnosed with chronic migraine; (ii) have migraine headaches for an average of four or more days per month for at least three months prior to the start of treatment with this drug; (iii) be already receiving non-pharmacological treatment such as sleep and dietary guidance, maintenance of proper weight, stress management, and acute treatment of migraine attacks, etc., and having difficulty in daily life even if these treatments are appropriately administered; and (iv) the use or continuation of any of the migraine attack onset suppressants approved in Japan (propranolol hydrochloride, sodium valproate, lomerizine hydrochloride, etc.) must not be possible due to one or more of the following reasons: (a) insufficient efficacy, (b) insufficiently tolerable, or (c) presence of contraindications or strong safety concerns in terms of side effects, etc. The headache and TN diagnoses were based on the International Classifications of Headache Disorder, 3rd edition [15]. During the three-month erenumab use, previous medications were continued.

Clinical variables and outcomes

We collected patients' characteristics, such as age, sex, onset age, region and laterality of TN, Barrow Neurological Institute Pain Intensity Score before erenumab treatment, and previous prophylactic medication for TN and migraine. We used clinical data from headache diaries that were kept on paper or electronically. Monthly headache days (MHD) were defined as the monthly values over the respective observation period of 30 days. The outcomes were defined as the changes in the numerical rating scale (NRS) of TN and MHD before treatment (0 months), one month, and three months. One month means one to four weeks, and three months means 9-12 weeks after the start of treatment. The period to realize the erenumab effect for TN and the continuation period of erenumab were also investigated.

Statistical analysis

Results were shown as the median (range). Shapiro-Wilk test confirmed the normal distribution. Paired t-tests or Wilcoxon's test was performed appropriately to compare NRS and MHD before and after treatment. We conducted these analyses using IBM SPSS Statistics for Windows, Version 28.0 (Released 2021; IBM Corp., Armonk, New York, United States). A two-tailed p-value of <0.05 was considered statistically significant.

## Results

**Figure 1:**
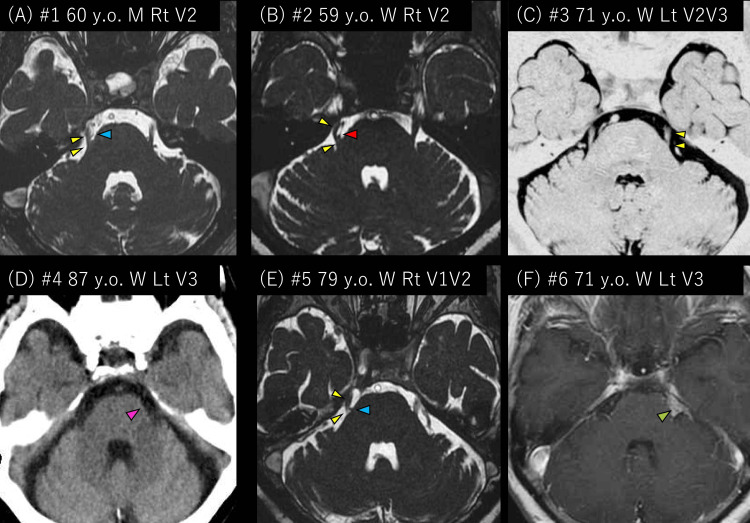
Radiological findings of the six trigeminal neuralgia patients. (A) Patient 1. The right trigeminal nerve (yellow arrowheads) was compressed by the petrosal vein (blue arrowhead). (B) Patient 2. The right trigeminal nerve (yellow arrowheads) was compressed by the superior cerebellar artery (red arrowhead). (C) Patient 3. No structures compressed the left trigeminal nerve (yellow arrowheads). (D) Patient 4. No structures were found at the left cerebellopontine cistern on the computed tomography (pink arrowheads). (E) Patient 5. The right trigeminal nerve (yellow arrowheads) was compressed by the petrosal vein (blue arrowhead). (F) Patient 6. The gadolinium-enhanced tumor was found at the left cerebellopontine cistern (green arrowhead).

The median age of the six patients was 71 years (59-87). Five females and one male were included. All six patients had episodic migraine. Three had TN due to vessels, one had secondary TN due to a tumor, and one had TN without neurovascular compression. The etiology of the TN of Patient 4 was unknown because she could not undergo magnetic resonance imaging due to a pacemaker. She also had an allergy to iodinated contrast medium Patients 1, 2, 3, and 6 were suggested surgical treatments, but they did not want them, considering the complications. Patients 4 and 5 were considered inoperable due to old age. The radiological findings of the six patients are shown in Figure 1. Other details are described in Table 1.

**Table 1:** Patients characteristics MHD; monthly headache days; MRI; magnetic resonance imaging, NRS; numerical rating scale, TN; trigeminal neuralgia.

No.	Sex	Age (years)	Onset age (years)	Region	Laterality	TN types	Barrow Neurological Institute Pain Intensity Score	Medication	NRS	MHD	Period to realize the effect	1 month NRS	1 month MHD	3 month NRS	3 month MHD	Continuation period
1	M	60	48	V2	R	Classical due to petrosal vein.	4	Carbamazepine 600mg, goreisan 7.5g.	8	4	Not effective.	8	4	-	-	1 month, stopped.
2	F	59	57	V2	R	Classical due to superior cerebellar artery.	5	Pregabalin 75mg, goreisan 7.5g. Rash due to carbamazepine.	10	4	2.5 months	10	4	5	1	3 months, stoppped, operation
3	F	71	63	V2, V3	L	Idiopathic.	4	Valproic acid 300 mg, goreisan 7.5 g. Rash due to carbamazepine.	7	5	2 weeks	2	0	0	1	3 months, continued.
4	F	87	60	V3	L	MRI could not be performed due to pacemaker.	3	Carbamazepine 400 mg, pregabalin 50 mg, goreisan 7.5 g.	8	10	2 weeks	0	1	0	1	6 months, continued.
5	F	79	59	V1, V2	R	Classical due to petrosal vein.	3	Carbamazepine 200 mg, goreisan 7.5g.	10	4	2 weeks	5	4	4	2	3 months, continued.
6	F	71	62	V3	L	Secondary due to tumor.	4	Carbamazepine 600 mg, pregabalin 50 mg, goreisan 7.5 g.	8	4	1 week	2	1	-	-	2 months, continued.

**Figure 2:**
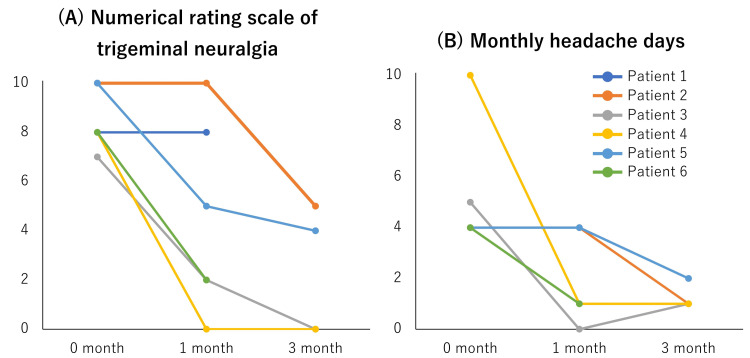
Treatment efficacy Chronological changes of a numerical rating scale of trigeminal neuralgia (A) and monthly headache days (B) after starting erenumab treatment.

Patient 1 stopped erenumab because he did not feel any efficacy; his MHD did not improve from four days/month. Patient 6 took erenumab for two months, but we had not yet asked about the efficacy of the three months. The other four patients continued erenumab use. There were no side effects of erenumab. Five (83%) of the six patients reported improved NRS of TN. The median NRS of TN before, one, and three months after treatment were 8 (7-10), 3.5 (0-10), and 2 (0-5, n=4) (Figure 2A), and the median MHD was 4 (4-10), 2.5 (0-4), and 1 (1-2, n=4) (Figure 2B).

There was a statistically significant improvement in NRS before treatment and after one month of treatment (p = 0.031), or NRS before treatment and after three months of treatment (p = 0.002). There was no significant reduction of MHD before treatment and after one month of treatment (p = 0.109), or NRS before treatment and after three months of treatment (p = 0.285).

## Discussion

We studied six migraine patients with comorbid TN treated with 70 mg of erenumab. Five (83%) of the six patients reported improved NRS of TN. This is the third report on erenumab for TN.

Drug treatment for TN is performed with carbamazepine as the first choice; it is effective for 90% of TN patients for pain control. However, the effect may not be sustained in the long term, and adverse effects sometimes lead to withdrawal in up to 40%. Carbamazepine is the drug indicated for TN. Lamotrigine, baclofen, pregabalin, gabapentin, phenytoin, and botulinum toxin type A are considered other drugs, but there is very less supportive evidence. When the medical treatment fails, surgical treatment or radiosurgery is considered. The one-year response rate for microvascular decompression is 68-84%, and the five-year response rate is 61-80%. For radiosurgery, they are 58-71% and 33-56%, respectively. Neuromodulation is also considered when these treatments fail [2]. Thus, there is still no treatment to control TN completely. It may also become uncontrollable with age, and further treatments are still desired.

Parascandolo first reported 10 patients of TN in 2021, and nine of them improved their NRS of TN after six months of erenumab treatment [9]. The dosage of erenumab was not described. Five of them reported being pain-free at six months. The median age was 51 years (44-64). Seven females and three males were included and five of the 10 had headache disorders. One patient with insufficient efficacy was a 44-year-old male with hypertension, cholecystectomy, fibromyalgia, anxiety, and depression, with highly frequent headaches at 25 days per month.

Andersen reported in 2022 the first randomized, double-blind, placebo-controlled trial to evaluate the effect of erenumab in patients with TN [10]. The study suggested that erenumab is ineffective in reducing both pain intensity and the frequency of pain paroxysms in TN compared with a placebo. Eighty participants aged 18 to 85 years old were randomly assigned to erenumab 140 mg (n=40) or placebo (n=40). At four weeks, there was no difference between groups in the intention-to-treat population's response rate (14 (35%) of 40 with erenumab vs. 18 (45%) of 40 with placebo). Of the 40 individuals, 20 (50%) in each group reported adverse events. Constipation (28%) and headache (10%) were the most frequent side effects in the erenumab group, while headache (13%), constipation (10%), and abdominal pain (10%) were most frequent in the placebo group.

The former report seemed similar to ours. Significantly, the effect was observed even in TN without migraine. The latter was a negative report regarding erenumab for TN without migraine. However, the study was underpowered because the placebo response was 40%, more than double compared to the 20% used in the ad-hoc sample size calculation. Compared to these reports, our patients all had both migraine and TN treated with 70 mg of erenumab, not 140 mg. Two of the six were inoperable. Considering the heterogeneity of the patients' characteristics in the three studies, we believe that the efficacy of erenumab for TN is not necessarily negative.

Our preliminary results, combined with the previous report [9], suggested that erenumab can be an alternative medicine for TN. A convenient dosing schedule of percutaneous injection every 28 days and a favorable side effect profile can be superior to other existing treatments. It may be effective in elderly patients who cannot be operated on and in TN without neurovascular compression, in other words, with no identifiable cause on radiological imaging [16]. In the course of our migraine awareness activities [17] and epidemiological studies [18-20], we sometimes encounter TN patients who actually have migraine headaches, as described above. Further real-world data accumulation and prospective randomized-control trials for patients with both TN and migraine using 70 mg erenumab are expected.

As a limitation, these results were based on a small case series at a single hospital in Japan. Therefore, whether the results will be similar for migraine and comorbid TN patients remains unknown. Our findings were preliminary, and a bigger sample size is required for this study to draw more firm conclusions. The patients may have started keeping headache diaries and paying closer attention to their health after receiving erenumab injections. Other preventative medications might possibly have had a delayed efficacy. The possibility of placebo effects could not be eliminated in this small case series. Also, the preventive effect of erenumab for migraine is not clarified in this cohort. Finally, stereotactic radiosurgery [21] and percutaneous rhizotomy [22] have proven effective for TN in patients who cannot or are unwilling to undergo surgery with craniotomy. However, we think that these treatments are more invasive compared to erenumab injection.

## Conclusions

We described six migraine patients with comorbid TN treated with 70 mg of erenumab. They had had at least one type of prophylactic medication for migraine before erenumab use, but it failed. Five (83%) of the six patients reported improved NRS of TN after erenumab injection. Our findings were preliminary, and a bigger sample size is required for this study to draw stronger conclusions. However, it is possible, although rare, that there are migraine patients in whom the NRS of comorbid TN improves with the use of erenumab, an anti-CGRP receptor monoclonal antibody.
